# A matched-pair cluster design study protocol to evaluate implementation of the Canadian C-spine rule in hospital emergency departments: Phase III

**DOI:** 10.1186/1748-5908-2-4

**Published:** 2007-02-08

**Authors:** Ian G Stiell, Jeremy Grimshaw, George A Wells, Doug Coyle, Howard J Lesiuk, Brian H Rowe, Robert J Brison, Michael John Schull, Jacques Lee, Catherine M Clement

**Affiliations:** 1 Department of Emergency Medicine, University of Ottawa, Ottawa, Canada; 2 Clinical Epidemiology Program, Ottawa Health Research Institute Ottawa, Ottawa, Canada; 3 Department of Medicine, University of Ottawa, Ottawa, Canada; 4 Divison of Neurosurgery, University of Ottawa, Ottawa, Canada; 5 Department of Emergency Medicine, University of Alberta, Edmonton, Canada; 6 Department of Emergency Medicine, Queen's University, Kingston, Canada; 7 Division of Emergency Medicine, University of Toronto, Toronto, Canada

## Abstract

**Background:**

Physicians in Canadian emergency departments (EDs) annually treat 185,000 alert and stable trauma victims who are at risk for cervical spine (C-spine) injury. However, only 0.9% of these patients have suffered a cervical spine fracture. Current use of radiography is not efficient. The Canadian C-Spine Rule is designed to allow physicians to be more selective and accurate in ordering C-spine radiography, and to rapidly clear the C-spine without the need for radiography in many patients. The goal of this phase III study is to evaluate the effectiveness of an active strategy to implement the Canadian C-Spine Rule into physician practice. Specific objectives are to: 1) determine clinical impact, 2) determine sustainability, 3) evaluate performance, and 4) conduct an economic evaluation.

**Methods:**

We propose a matched-pair cluster design study that compares outcomes during three consecutive 12-months "before," "after," and "decay" periods at six pairs of "intervention" and "control" sites. These 12 hospital ED sites will be stratified as "teaching" or "community" hospitals, matched according to baseline C-spine radiography ordering rates, and then allocated within each pair to either intervention or control groups. During the "after" period at the intervention sites, simple and inexpensive strategies will be employed to actively implement the Canadian C-Spine Rule. The following outcomes will be assessed: 1) measures of clinical impact, 2) performance of the Canadian C-Spine Rule, and 3) economic measures. During the 12-month "decay" period, implementation strategies will continue, allowing us to evaluate the sustainability of the effect. We estimate a sample size of 4,800 patients in each period in order to have adequate power to evaluate the main outcomes.

**Discussion:**

Phase I successfully derived the Canadian C-Spine Rule and phase II confirmed the accuracy and safety of the rule, hence, the potential for physicians to improve care. What remains unknown is the actual change in clinical behaviors that can be affected by implementation of the Canadian C-Spine Rule, and whether implementation can be achieved with simple and inexpensive measures. We believe that the Canadian C-Spine Rule has the potential to significantly reduce health care costs and improve the efficiency of patient flow in busy Canadian EDs.

## Background

### Introduction

Physicians in Canadian emergency departments (EDs) annually treat 185,000 alert and stable trauma victims who are at risk for cervical spine (C-spine) injury (CSI). However, only 0.9% of these patients have suffered a cervical spine fracture. Current use of radiography is not efficient. More than 98% of C-spine radiographs are negative, and there is considerable variation among hospitals and physicians in radiography use. C-spine radiographs are "little ticket" items, low cost procedures that significantly add to health care costs due to high volume. In addition, alert and stable trauma patients often are immobilized on a backboard with a rigid collar and sandbags for many hours. This leads to considerable patient discomfort and to unnecessary use of valuable time and space in our crowded EDs.

A clinical decision rule is derived from original research, and is defined as a decision-making tool that incorporates three or more variables from the history, examination, or simple tests. These rules help clinicians with diagnostic or therapeutic decisions at the bedside. We previously developed decision rules to allow more selective use of radiography for patients with ankle [1-4] and knee injuries [5-7].

**Figure 1:**
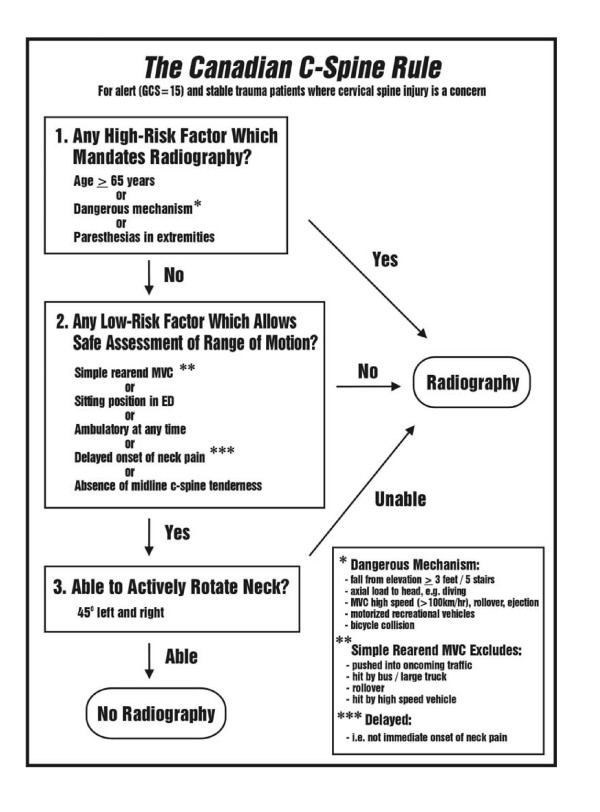
The Canadian C-Spine Rule.

This protocol builds on previous funded grants to determine feasibility [8], (phase 0; 1995–96; N = 6,855), to develop a clinical decision rule for cervical spine radiography [9] (phase I; 1996–99; N = 8,924), and to prospectively validate this "Canadian C-Spine Rule" (phase II; 1999–2002; N = 8,283). The Canadian C-Spine Rule is comprised of simple clinical variables (Figure 1), and is designed to allow physicians to be much more selective and accurate in ordering cervical spine radiography and to rapidly clear the C-spine without the need for radiography in many patients. In the multicentre prospective validation (phase II), we studied 8,283 patients and confirmed the accuracy and reliability of the rule, as well as the potential to significantly reduce radiography and improve patient flow in our crowded EDs.

The goal of the current protocol (phase III) is to evaluate the effectiveness of an active strategy to implement the Canadian C-Spine Rule into physician practice in multiple EDs. We wish to test both the impact of the rule and the effectiveness of an implementation strategy that is inexpensive and easy to adopt. In other words, we wish to determine whether the Canadian C-Spine Rule can actually be adopted into clinical practice and whether the efficiency of patient care can be improved. Secondary objectives are to determine the sustainability of the intervention, to further evaluate the accuracy of the rule, and to conduct an economic evaluation of the potential for cost savings.

### Clinical decision rules

**Figure 2:**
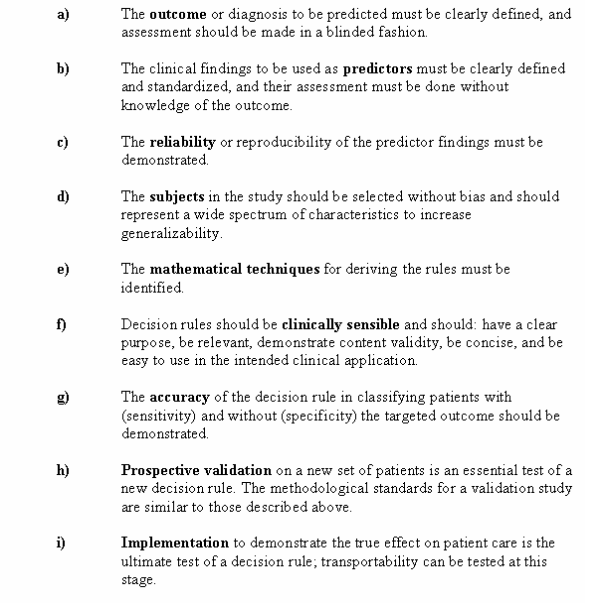
Methodological Standards for Clinical Decision Rules.

Clinical decision (or prediction) rules help to reduce the uncertainty of medical decision-making by standardizing the collection and interpretation of clinical data [10-13]. A decision rule is derived from original research, and may be defined as a decision-making tool that incorporates three or more variables from the history, physical examination, or simple tests. These decision rules help clinicians with bedside diagnostic or therapeutic decisions. To fully develop a clinically effective rule is a lengthy process that involves separate studies to derive, prospectively validate, and finally implement the rule. The methodological standards for the derivation and validation of decision rules are summarized in Figure 2[14-17].

Implementation to demonstrate the true effect on patient care is the ultimate test of a decision rule [18]. Unfortunately, many clinical decision rules are not prospectively assessed to determine their accuracy, reliability, clinical sensibility, or potential impact on practice. This evaluation is critical because many statistically derived rules or guidelines fail to perform well when tested in a new population [19-21]. The reason for this performance failure may be statistical, such as over-fitting or instability of the original derived model [22], or may be due to differences in prevalence of disease, or in how the decision rule is applied [23,24]. Most decision rules are never used after derivation because they are not adequately tested in validation or implementation studies [25-27].

### Dissemination and uptake of new health care information

One of the most consistent findings in health services research is the uneven uptake of research across different healthcare settings, countries and specialties. Recognition of failure of traditional dissemination approaches has led to greater policy and research interest into the effectiveness and efficiency of different dissemination and implementation strategies. For example, several large studies in multiple cities have clearly demonstrated the effectiveness of implementing the Ottawa Ankle Rules [3,4,28]. However, at least one study found no impact from the rules with a dissemination strategy that relied upon a single lecture given at each hospital by a visiting speaker [29].

There is a growing body of rigorous evaluations of different dissemination and implementation strategies [30-32]. Grimshaw undertook an overview of 41 systematic reviews of professional behavior change strategies [33]. This included one systematic review that specifically considered test ordering [34]. These systematic reviews identified a variety of dissemination and implementation strategies that are effective under certain conditions, but current knowledge is imperfect. Passive dissemination (i.e., mailing educational materials to targeted clinicians) is generally ineffective and is unlikely to result in behavior change when used alone. However, this approach may be useful for raising awareness of the desired behavior change. Active approaches are more likely to be effective, but also likely to be more costly. Interventions of variable effectiveness include audit and feedback and use of local opinion leaders. Generally effective strategies include educational outreach (for prescribing behavior) and reminders.

In addition, Grimshaw has completed a systematic review of rigorous evaluations of guideline dissemination and implementation strategies [35]. In all, 235 studies reporting 309 comparisons met the inclusion criteria. The overall quality of the studies was poor. The majority of interventions observed modest improvements in care, with median absolute improvements ranging from 6.0% to 13.1%.

Another important issue is sustainability. Implementation studies are often criticized because effects of the intervention are only transient, and followed by significant decay. For example, Fowkes and colleagues observed decay effects amongst four interventions to improve radiology referral in in-patient settings over 12 months [36]. In an overview of interrupted time series designs in implementation research, Ramsay found that often there was a step improvement in care followed by a decay effect [37].

The results of these systematic reviews highlight the imperfect evidence base currently available to support decisions about which dissemination and implementation strategies are most likely to be efficient under different circumstances. Grimshaw and colleagues called for further rigorous evaluations of the effectiveness and efficiency of different dissemination and implementation interventions.

### Previous cervical spine work by authors

#### Feasibility studies

In 1994 and 1995, a research formulation workshop and a funded pilot study were conducted to evaluate current practice patterns, and this demonstrated very large variation across Canada in the use of cervical spine radiography [8]. Two mail surveys of the attitudes of emergency physicians toward decision rules also were conducted. Survey results revealed that 98% of Canadian physicians would consider using a sensitive and reliable clinical decision rule for the use of cervical spine radiography [38]. An international survey found that the majority of physicians indicated very strong support for a cervical spine radiography decision rule [39].

#### Results of phase I: derivation

The results of phase I, the derivation of the Canadian C-Spine Rule (Figure 1), were published in *JAMA *in October 2001 [9]. In this prospective cohort study, physicians evaluated patients for 20 standardized clinical findings prior to radiography. Among the study sample, 151 (1.7%) had important C-spine injury. The resultant model and final Canadian C-Spine Rule stratifies patients into high-, medium-, and low-risk groups, and requires evaluation of active range-of-motion for those in the low-risk group. This rule was cross-validated on the derivation sample (N = 8,924) and was found to identify all 151 cases of clinically important CSI, with a sensitivity of 100% (95% CI 98–100). The rule also performed with a specificity of 42.5% and would have required radiography for only 58.2% of patients, a 23.9% relative reduction from the current ordering rate of 76.5%.

#### Results of phase II: prospective validation

The results of phase II, the Canadian C-Spine Rule validation study, were published in 2003 [40].

#### Objectives

The principal objectives of phase II (1999–2002) were to prospectively assess the accuracy, reliability, and clinical sensibility of the Canadian C-Spine Rule and the United States (U.S.) based National Emergency X-Radiography Utilization Study (NEXUS) low-risk criteria in a new set of alert and stable trauma patients. The NEXUS criteria include five items and was first described in 1992 [41], subsequently validated in a study in the U.S, involving 34,069 trauma patients [42,43].

#### Summary of methods

This prospective cohort study was conducted in the emergency departments of nine Canadian tertiary care hospitals. The Canadian C-Spine Rule and NEXUS criteria were interpreted by 394 physicians for patients before radiography. A second physician independently assessed some patients for the same criteria when feasible, and inter-observer agreement was determined. The primary outcome, clinically important CSI, was evaluated after the clinical assessment by standard plain radiography of the cervical spine, optional flexion-extension views, and CT, if clinically indicated.

## Results

In all, 8,283 patients were included in the final analysis [44]. Among all the patients, 169 (2.0%) had clinically important CSI. In 845 patients (10.2%), physicians did not evaluate range of motion, as required by the Canadian C-Spine Rule, and were categorized as indeterminate cases. Seven of these 845 patients had clinically important CSI. In the analysis that excluded the indeterminate cases, the Canadian C-Spine Rule was more sensitive than the NEXUS criteria (99.4% vs. 90.7%, P < 0.001), and more specific for injury (45.1% vs. 36.8%, P < 0.001). The kappa value for inter-observer agreement in the interpretation of the rules in 142 cases was 0.63 for the Canadian C-Spine Rule (95% CI 0.49 – 0.77) and 0.47 for the NEXUS criteria (95% CI 0.28 – 0.65). Also, the use of the Canadian C-Spine Rule would have resulted in lower radiography rates compared to the use of the NEXUS criteria (55.9% vs. 66.6%, P < 0.001). The potential impact on ED crowding also was assessed by determining the mean length-of-stay in the ED for patients without injury. Results revealed that patients who did not undergo radiography spent almost two hours less time in the ED (123.2 min vs. 232.9 min, P < 0.001) than did patients who had radiography.

### Summary of findings

We found the Canadian C-Spine Rule to be highly sensitive for clinically important CSI, identifying 161 of 162 cases. In the combined phases I and II, the rule would have identified 312 of 313 CSI cases, a sensitivity of 99.7% (95% CI 98–100). We also found the rule to very reliable with a kappa value of 0.63. Implementation of the Canadian C-Spine Rule would be expected to lead to much more rapid, yet safe, clearing of the cervical spine for alert patients with trauma who are in stable condition, and hence, more rapid flow of trauma patients through our crowded EDs.

### Rationale for the study

#### Potential benefits

What are the potential implications of a decision rule for the use of cervical spine radiography in alert and stable trauma patients? First, patient care will be standardized and improved. The considerable variation in current Canadian practice suggests the need for accurate and reliable guidelines. Patients will no longer undergo unnecessary radiography or prolonged immobilization. Second, ED overcrowding will be aided by the ability of MDs to quickly and clinically clear the cervical spine of stable trauma patients without the need for complete radiography. Rather than waiting hours in a resuscitation bay on a backboard, patients can be sent to less acute areas in the ED without immobilization – or can be sent home promptly. Third, health care system savings will be an important benefit in this era of severe fiscal pressures on our hospitals. Both the current variation in practice and the very low yield of cervical spine radiography for alert stable trauma patients suggest significant potential for reducing the use of radiography. Our previous studies in multiple Canadian hospitals showed large reductions in the use of ankle and knee radiography after the implementation of our Ottawa Ankle Rules and the Ottawa Knee Rule [3,4,7]. We estimate that a 25% to 50% relative reduction in the use of cervical spine radiography could be safely achieved with effective implementation of the Canadian C-Spine Rule.

#### Implementation study

Why do we need to conduct this proposed phase III implementation study, especially after the large and successful phase I derivation and phase II validation studies? First, physician behaviour change is not a certainty because CSI is a much more serious condition than ankle or knee injuries. Physicians may not prove to be as compliant with the Canadian C-Spine Rule as they have been with the Ottawa Ankle and Knee Rules. Phase II demonstrated problems with compliance and with accuracy of interpretation. We need to evaluate the real savings that can be achieved as opposed to the potential savings. Second, efficient and pragmatic methods to affect implementation of clinical guidelines are required. Our previous implementation studies for the ankle and knee rules used a wide range of strategies, many of which were expensive and not practical for everyday use. In this study, we propose to use implementation strategies that are simple and inexpensive, and which any hospital could easily adopt on a permanent basis. Third, sustainability is often a weakness of elaborate implementation strategies. We will determine whether our approach leads to sustained effects, or if there is decay.

#### Specific objectives

The goal of phase III is to evaluate the effectiveness and safety of an active strategy to implement the Canadian C-Spine Rule into physician practice in multiple EDs, compared to a control strategy that relies upon passive measures. Specific objectives are to:

*Determine clinical impact *by comparing the intervention and control sites, individually and collectively, during the "before" and "after" periods for:

a) Cervical spine radiography rates, such as proportion of potential injury patients referred for radiography; this is the primary study objective;

b) Number of missed CSI, such as clinically important CSI not identified during initial ED visit;

c) Number of serious adverse outcomes, such as development of neurological deficit after initial assessment in ED;

d) Length of stay in the ED, such as the time from arrival until discharge; and

e) Patient satisfaction with ED care, particularly when cervical spine radiography is not ordered.

*Determine sustainability *of clinical impact by comparing the intervention and control sites, individually and collectively, during the "after" and "decay" periods for objectives a)-d) above.

*Evaluate performance *of the Canadian C-Spine Rule, during the "after" period at the intervention sites:

a) Accuracy of the rule, such as sensitivity and specificity for identifying clinically important CSI;

b) Physician accuracy in interpretation of the rule; and

c) Physician comfort and compliance with use.

*Conduct an economic evaluation *to determine the potential for cost savings with widespread implementation of the rule.

## Methods

### Design

**Figure 3:**
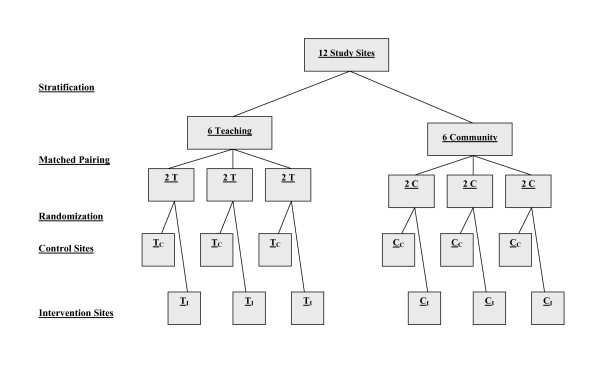
Matched-Pair Design Allocation Scheme for "After" Period.

We propose to conduct a matched-pair cluster design study that compares outcome measures during three consecutive 12-month "before," "after," and "decay" periods at six pairs of "intervention" and "control" sites (Figure 3) [45]. These 12 hospital ED sites will be stratified by the classification of "teaching" or "community" hospital, and matched according to baseline cervical spine radiography ordering rates during the "before" periods. Using computer-generated numbers, sites within each pair will be randomly allocated to either intervention or control groups by our senior biostatistician. During the "after" period at the control sites, there will be no specific implementation strategies, and physicians will order radiography according to personal judgment. During the "after" period at the intervention sites, strategies will be employed to actively implement the Canadian C-Spine Rule into physician practice. This "after" period will evaluate the time to full effect, as well as maximum effect of the implementation. During the third 12-month period – the "decay" period – implementation strategies will continue as in the "after" period. This will allow us to evaluate the sustainability of the effect of implementation, such as whether our simple and inexpensive implementation strategy can be expected to have a long-term effect, or whether there will be significant decay. Due to the nature of this intervention, blinding will not be possible.

### Study population

#### Inclusion criteria

All alert and stable adults presenting to the study hospital EDs after sustaining acute blunt trauma to the head or neck will be eligible, and consecutive eligible trauma patients will be entered into the study. Patient eligibility will be determined based on these criteria at the time of arrival in the ED.

"Trauma to the head and neck" will include patients with either: i) neck pain with any mechanism of injury (subjective complaint by the patient of any pain in the posterior midline or posterolateral aspect of the neck); or ii) no neck pain, but all of the following: some visible injury above the clavicles, has not been ambulatory, and associated with a high-risk mechanism of injury (i.e., motor vehicle collision including motorcycle, pedestrian struck by a motor vehicle, bicycle collision, fall greater than or equal to 3 feet or 5 steps, diving, or contact sport with axial load to head and neck).

"Alert" is defined as a Glasgow Coma Scale [46] score of 15 (converses, fully oriented, and follows commands).

"Stable" refers to normal vital signs as defined by the Revised Trauma Score [47] (systolic blood pressure 90 mm Hg or greater, and respiratory rate between 10 and 24 breaths per minute).

"Acute" refers to injury within the past 48 hours.

#### Exclusion criteria

Exclusion criteria include: a) patients under the age of 16 years; b) patients who do not satisfy the definition of "trauma to the head and neck" as defined above (e.g., patients with neither neck pain nor visible injuries above the clavicles will be excluded); c) patients with Glasgow Coma Scale score less than 15; d) patients with unstable vital signs (systolic BP < 90; respiratory rate less than 10 or more than 24); e) patients whose injury occurred more than 48 hours previously; f) patients with penetrating trauma from stabbing or gunshot wound; g) patients with acute paralysis (paraplegia, quadriplegia); h) patients with known vertebral disease (ankylosing spondylitis, rheumatoid arthritis, spinal stenosis, or previous cervical spine surgery); or i) patients who return for reassessment of the same injury.

#### Patient safety

We are convinced that the use of the Canadian C-Spine Rule is accurate and reliable, and that the proposed study will respect patient safety at all times. Use of the rule will be encouraged, but the decision to order radiography will always be at the discretion of the attending physician, as it is at present. Physicians will know that they can "override" the rule at any time when they have concerns about patient welfare. The Canadian C-Spine Rule has proven to be very sensitive in identifying CSI and, in fact, one could argue that the rule is more accurate than Canadian emergency physicians. We do know that, in current Canadian practice without the rule, patients are being discharged from the ED with undiagnosed fractures. We expect this occurrence to be less frequent in the proposed study when the rule is available as a guide.

### Ethical considerations

All the respective research ethics boards have approved the study without the need for informed patient consent at the time of the ED visit. During a particular period in time at a given site, all eligible patients will be managed by the physicians in the same manner, because the unit of study allocation is the hospital, not the patient. As is typical of cluster allocated, matched-pair design studies, individual patients will not be randomized and physicians will order cervical spine radiography in a similar fashion for all patients at their site [48]. Patients will not be subjected to new therapy, invasive procedures, undue risk or discomfort, or use of diagnostic radiography beyond that which would normally be required in the course of patient care. Physicians will be encouraged to use the Canadian C-Spine Rule as a guide for ordering radiography, but will ultimately base their decision on their own judgment as to what is the safest way to manage each individual patient. We note that Canadian physicians are already selective in ordering C-spine radiography, and omitted radiography for 28.3% of cases in phase II. At the same time, we know that the physicians missed some fractures. Patient confidentiality will be maintained throughout the study, and patient names will be removed from all records. The small numbers of patients who are selected for follow-up telephone interviews will have an opportunity to give verbal consent to the ED registered nurse who makes the call. This is consistent with the approach approved by the research ethics boards for follow-up in phases I and II. The safety of the study will be overseen by an independent data monitoring safety board, comprised of a biostatistician, an emergency physician, and a neurosurgeon. This board will have the mandate to terminate the study at any time should there be concerns about adverse patient outcomes.

### Setting

The study setting will be six "teaching" and six large "community" hospital EDs, with a combined annual ED volume of approximately 670,000 patient visits. We believe that the generalizability of our findings will be greatly enhanced by including both teaching and community hospitals from a variety of cities (population range 30,000 to 4,000,000) in different areas of Canada. We define a "teaching" hospital as one that is a core educational institution for a medical school's undergraduate and postgraduate students, and whose hospital staff physicians have full-time appointments to that medical school. "Community" hospitals may provide experience for some medical trainees, but the majority of patient care is provided by staff physicians who do not have fulltime appointments with a medical school.

### Study interventions

#### Control sites

No specific interventions will be undertaken to alter the cervical spine radiography ordering behavior of the ED physicians. These sites will exemplify the impact of "diffusion" of new medical information. The Canadian C-Spine Rule will be familiar to some clinicians because of the publication of our phase I results in *JAMA *in October 2001, as well as scientific presentations at national meetings in Canada and the U.S. and a few presentations at continuing education meetings in Canada.

#### Intervention sites

We intend to pursue simple and inexpensive strategies to actively implement the use of the Canadian C-Spine Rule at the intervention sites. Therefore, we have designed an intervention that we consider is deliverable throughout Canadian settings with few additional resources.

#### Details of planned interventions

Each ED physician group will be asked to discuss and agree to a policy of ordering cervical spine radiography for alert and stable trauma patients according to the Canadian C-Spine Rule. Minor educational initiatives for the ED physicians will include the distribution of manuscripts, pocket cards, and posters, as well as a single one-hour teaching session to review the evidence and clinical application of the Canadian C-Spine Rule. The ED and Radiology departments will collaborate to institute a process-of-care modification with a mandatory "online" reminder of the Canadian C-Spine Rule at the point of requisition for cervical spine radiography. All cervical spine radiography ordered in the ED will require that the ordering physician complete a special paper or computer-based requisition that includes the Canadian C-Spine Rule algorithm criteria. The physician must "check off" the criteria, or the radiology department will not process the request. The physician may override the rule, and order radiography according to his/her clinical judgment, but will be asked to indicate the reason. Those sites that use paper requisitions will implement a new pad of special cervical spine radiography requisitions. Those sites that order radiography by computer will have an on-screen version of the rule made available by software modification.

#### Rationale for choice of intervention

We have designed our intervention based upon theoretical considerations, currently available evidence, and discussions with collaborators. The theory of planned behavior proposes that behavior is determined by the individual's intentions to engage in a behavior, and the degree of control they feel they have over that behavior. Intention strength is determined by three variables: attitudes toward the behavior, subjective norms, and perceived behavioral control [49]. ED physicians' intentions to use the Canadian C-Spine Rule would be weak if they were not convinced that the rule would reduce unnecessary x-rays, or if they thought that it was unimportant to reduce unnecessary x-rays (attitudes to the behavior), if they believed that important colleagues did not think that it was important to follow the C-spine rules (subjective norms), or if they did not think that it was possible to follow the rules (perceived behavioral controls). It is recognized increasingly that other factors (i.e., problems of information processing in busy clinical surroundings) intervene between intentions and behaviors that could result in failure to follow the C-spine rules, even if the physician intends to do so [50]. Our interventions will target these different barriers. The educational interventions will target physicians' attitudes toward the C-spine rules. The local consensus process will target physicians' subjective norms by getting buy-in from all the local key stakeholders. The mandatory online reminder will prompt physicians to follow the rule, if they are considering radiography in alert and stable trauma patients.

Empirical evidence for our choice of intervention is available from the review by Solomon and colleagues [34]. They suggest that local consensus processes predisposes to behavior change, especially if coupled with system changes. They also note that the combinations of educational and system changes are more likely to lead to improvements in test ordering. Grimshaw and colleagues conclude that "Reminders are the intervention that have been evaluated most ... [and]...are a potentially effective intervention ... likely to result in moderate improvements in process of care". Further, the use of obligatory reminders appears more successful than voluntary reminders [51]. In discussion with our collaborators, these interventions appeared to be achievable and had face validity.

### Outcome measures and data collection

#### Measures of clinical impact

The following will be collected at both the intervention and control sites during all three study periods by dedicated study personnel who will review daily patient logs, ED patient records, radiology reports, and inpatient records.

Cervical spine radiography ordering proportions will be the primary study outcome, such as the proportion of eligible blunt trauma patients referred for plain cervical spine radiography during the initial ED visit. Daily patient census logs will be reviewed to identify potential injury patients, and then ED patient records (e.g., ambulance call reports, nursing notes, and physician notes) will be assessed to determine eligibility. Radiology reports and census lists will be used to determine if cervical spine radiography was performed.

*Number of missed CSI, such as number of clinically important CSI not identified during initial ED visit*. We validated the safety of the Canadian C-Spine Rule with detailed follow-up of patients in phase II. In order to significantly reduce the resources required for phase III, we propose not to specifically follow all patients who do not undergo radiography in the ED. Telephone follow-up of patients is very labor intensive and expensive. Rather, we propose to institute a strategy of surveillance to identify the uncommon occurrence of a fracture missed because no radiography was ordered. The more common missed fracture cases due to mis-reading of the radiograph will be identified through the normal radiology department quality assurance processes. ED patient visit logs at each study site will be monitored for 30 days to identify return visits by patients who do not undergo radiography during their ED visit. In addition, we will review the neurosurgery patient logs at all neurosurgical centers that are the traditional referral sites for the study hospitals. In many cases, the regional neurosurgical centers will be our study hospitals. Application of the same surveillance approach, regardless of phase or intervention group, minimizes the likelihood of bias. We recognize that there is a very small risk of not identifying a missed fracture, but feel that this approach is pragmatic and feasible.

*Number of serious adverse outcomes, such as development of neurological deficit after initial assessment in ED*. We will use the same surveillance approach described above for identifying missed fractures to identify the extremely rare occurrence of motor weakness and disability that develops after initial assessment in the ED.

*Length of stay in ED, such as the total length of stay from registration to discharge for patients who are neither admitted nor have a CSI*. This will be considerably impacted by the duration of cervical spine immobilization and radiography procedures.

Patient satisfaction with ED care will be determined by a random sample of 5% of "before" and "after" period patients (both those who did and those who did not receive radiography), who will be asked via telephone interview to rate their satisfaction on a five-point Likert scale at 30 days.

#### Sustainability of the intervention

The same clinical impact measures will be collected during the "decay" period to determine whether the effects achieved during the "after" period have been sustained.

#### Performance of the Canadian C-Spine Rule

This is a secondary study outcome. The rule will be evaluated during the "after" period at the intervention sites for those cases where physicians have completed the special study requisition and checked off the rule criteria. Rule criteria are:

*Accuracy of the rule, such as sensitivity and specificity for identifying clinically important CSI*. In interpretation of the rule, physician accuracy will be determined by comparing the physicians' notation on the radiography requisition to the "gold standard" interpretation of the rule made by the investigators' steering committee. Attention will be focused on fractures missed or potentially missed by physician misinterpretation.

*Physician comfort and compliance with use of the rule*. On the radiography requisition, physicians will be asked to indicate their comfort in following the rule for that specific patient, using a five-point Likert scale. When physicians choose not to follow the rule, they will be asked to indicate reasons for non-compliance.

#### Economic evaluation measures

The following will be evaluated for the economic impact of the C-spine rule: radiography rates after discharge will be determined by a random sample of 5% of "after" phase patients, who will be followed by telephone interview 30 days after the initial ED visit. This will ascertain if the patient has obtained cervical spine radiography through a family physician, clinic, or ED. We also will examine the length of stay in the ED (in hospital), if admitted, hospital admission for CSI (as opposed to other injuries), and operative repair of CSI.

### Data analysis

#### Measures of clinical impact

Every eligible patient who satisfies the inclusion and exclusion criteria during each of the three periods at all 12 sites will be included in the final analysis. No patient will be excluded due to non-compliance by the physicians or radiology departments. Sub-group analyses will evaluate teaching and community hospitals separately. Comparison of patient characteristics will be tested. All *p *values will be two-tailed. The primary analyses will compare the "before" and "after" periods. Secondary analyses will compare the "after" and "decay" periods in order to evaluate sustainability.

For the analysis of dichotomous data from this matched-pair design, a parametric approach will be used, based on the standard paired t-test (with *k*-1 = 5 degrees of freedom) to the differences in the event rates in the intervention and control site pairs. Although the assumptions of equal variances and approximate normality may not be satisfied, empirical studies suggest that this test procedure is robust to departures from these assumptions [52-54]. It is expected that the cluster sizes will be similar, but if they are highly variable, then a weighted t-test after transformation of the event rates to the logistic scale will be considered, as suggested by Donner and Donald [53]. Given the small number of pairs, exact procedures based on permutation tests also will be considered. Further, 95% confidence intervals will be calculated for the relative reductions in event rates. Similarly for the analysis of continuous data, the standard paired t-test (with *k*-1 = 5 degrees of freedom) to the differences in the mean response between the intervention and control site pairs will be used. If the relevant information from the previous period is available, the change from baseline for each cluster will be determined and used in the calculation of the difference in the event rate or response for each intervention and control site pair.

For each of the following clinical impact outcome variables, the change from the "before" to "after" periods in the proportions (or means) for each cluster will be determined, used in the calculation of the differences in the event rates (mean response) for each intervention and control site pair, and analyzed according to the above analysis plan. Clinical impact outcome variables include: cervical spine radiography ordering proportions, proportion of missed fractures, proportion of serious adverse outcomes, length of stay in ED in minutes and, patient satisfaction with the proportions indicating "very satisfied" or "satisfied."

#### Performance of the Canadian C-Spine Rule

Performance of the Canadian C-Spine Rule will be evaluated by the following measures.

Accuracy of the rule: The classification performance of the rule for clinically important CSI will be assessed with 95% CIs for sensitivity, specificity, negative predictive value, and positive predictive value. The "criterion interpretation" of the rule, such as positive or negative for CSI, will be made by the investigators based on the status of the patient for the component variables as documented by the physician.

Physician accuracy in interpretation of the rule will be calculated as the simple agreement between the physicians' notation on the requisition – to the investigators' "criterion interpretation" of the rule.

Physician comfort and compliance with use of the rule will be tabulated in a simple descriptive format.

#### Economic evaluation

We will adopt a decision analytic approach, whereby we will identify the incremental cost savings from both a health care sector and a societal perspective of the active strategy of implementation [55]. The model will consider two hypothetical cohorts of patients, a "usual practice" cohort, and a cohort where practice is as dictated through the active dissemination of the decision rule. Results will be generated through probabilistic analysis, as this is superior to simple deterministic analysis [56,57]. The principal resources in this analysis will be the costs of radiography and the associated patient time costs. In addition, the costs of settlement of litigation, the incremental costs of follow-up treatment due to missed fractures, and the costs of neurological deficits will be included. The design of the decision analytic model, sources of data, and analysis of uncertainty are provided in a supplemental file [58-64] [see Additional file 1].

### Sample size

#### Required patients

Refer to the supplemental file for more details on the sample size calculation [45,65-67] [see Additional file 2]. The study is based on a complex, stratified, matched-pair cluster design, such as a stratification factor type of hospital (teaching vs. community), a matched-pair (hospitals matched according to baseline cervical spine radiography), and cluster randomization (unit of randomization is the hospital and unit of analysis is the patient) [45,68]. Let k be the number of pairs needed to achieve power100(1-β)% for detecting a difference Δ in the cervical spine radiography event rates at the two-sided 100(1-α)% significance level. From the phase II study, the cervical spine radiography event rates for non-transfer patients at the participating sites ranged from 63.3% to 85.9%, with an average of 76% and an annual accrual per hospital of approximately 400 patients. We estimate that the between-cluster variance component is 0.00636, based on Gail et al (1992) for the COMMIT trial (i.e. *Var*(*d*_*j*_) = 0.0066125) [66]. It is expected that the event rate in the control group will not change. Based on a consensus of the participating site investigators, we believe that for the intervention group, a 15% relative decrease (or an absolute decrease of 11.4% from the baseline rate of 76%) in the event rate would be considered a minimal clinically important change (i.e. Δ = 0.114). Then for a significance level of 0.05 and power of 80%, *k *=(1.96+0.84)^2^(0.0066125)/.114^2 ^= 3.9891 and the number of matched-pairs required is (7/5)(3.9891) = 5.5847 where 7/5 adjusts for the small number of degrees of freedom [65]. Thus, six matched-pair clusters will be required with 400 patients per hospital for each of the ''before'' and ''after'' periods in the primary analysis. Because the exact benefits of stratifying by teaching and community hospitals in this matched-pair design are difficult to quantify, a conservative approach is adopted and the six matched-pair clusters will be selected [67]. Furthermore, we estimate that the sample size for the ''decay'' period will be the same as for the ''after'' period, and that this will allow sufficient power in the secondary analysis to identify complete decay of effect from the ''after'' to the ''decay'' period. In addition, this will provide a sufficient sample to calculate a precise estimate of the decay rate.

#### Feasibility and timing

Hence, we will require 4,800 eligible patients at the 12 sites for each of the three study periods, for a total of 14,400 patients. We plan three consecutive 12-month periods representing, respectively, the "before," "after," and "decay" periods. Based upon our knowledge of seven sites from phase II, and extrapolating from census information of five new sites, we expect no difficulty in achieving our sample size goals. Because all eligible patients are enrolled by default, and because no consent is required, we will not lose patients to physician non-compliance nor to patient refusal.

### Methodological issues

Why a matched-pair design? The matched-pair design is frequently used in community intervention studies and offers several advantages for studies like ours, in which the unit of allocation is the hospital rather than the patient. Matching on baseline data, such as radiography rates from the "before" period, helps prevent imbalance between the control and intervention groups. This design helps preserve power of analysis when relatively few (12) clusters are being studied.

Why three study periods? The "before" period will provide the baseline radiography rates, which will be the basis for the matching and the baseline for measuring change. The "after" period will measure the time to onset of the effect of the intervention, as well as the maximum effect. The "decay" period will allow us to evaluate the sustainability of the effect of implementation, such as whether our simple and inexpensive implementation strategy can be expected to have a long-term effect.

Will incorporation of phase II sites in phase III lead to contamination? Seven of the 12 sites participated in phase II, which will end at least six months prior to the onset of phase III. We believe there will be little or no carry-over effect on physician behavior in phase III because physicians did not apply the rule in phase II, but continued to order radiography according to their judgment. If anything, physicians ordered radiography at a higher rate (71.7%) during phase II than during the phase 0 baseline (58.0%).

## Discussion

### Relevance

Blunt trauma is a very common condition that is associated with excessive and variable use of radiography, and with prolonged periods of patient immobilization in the ED. The Canadian C-Spine Rule has been derived and validated in more than 16,000 patients, and would appear to have the potential to lead to important reductions in the use of radiography, health care savings, and diminished waiting times in our crowded EDs. However, many decision rules and guidelines have no impact on health care because of inadequate dissemination strategies. What we hope to demonstrate in the proposed phase III implementation study would affect an actual change in clinical behavior. We propose to evaluate the effectiveness of implementing the Canadian C-Spine Rule, and whether such implementation can be achieved with simple and inexpensive measures. We believe that the Canadian C-Spine Rule has the potential to significantly reduce health care costs and improve the efficiency of patient flow in busy Canadian EDs.

## Supplementary Material

Additional File 1 Details of Economic Analysis. The document provides a detailed description of the decision analytic model design as well as the sources of data and uncertainty. Click here for file

Additional File 2 Details of Sample size Calculation. The document provides a more detailed explanation of the variables and equation used to calculate the matched-pair cluster sample size. Click here for file
