# Supplementary material for: A matched-pair cluster design study protocol to evaluate implementation of the Canadian C-spine rule in hospital emergency departments: Phase III
Source: Implement Sci. 2007 Feb 8;2:4. doi: 10.1186/1748-5908-2-4 (PMC1802999; doi:10.1186/1748-5908-2-4)
Supplement: Additional File 1 — Details of Economic Analysis. The document provides a detailed description of the decision analytic model design as well as the sources of data and uncertainty. [file 1748-5908-2-4-S1.pdf]

Details of Economic Analysis

Structure of Decision Analytic Model

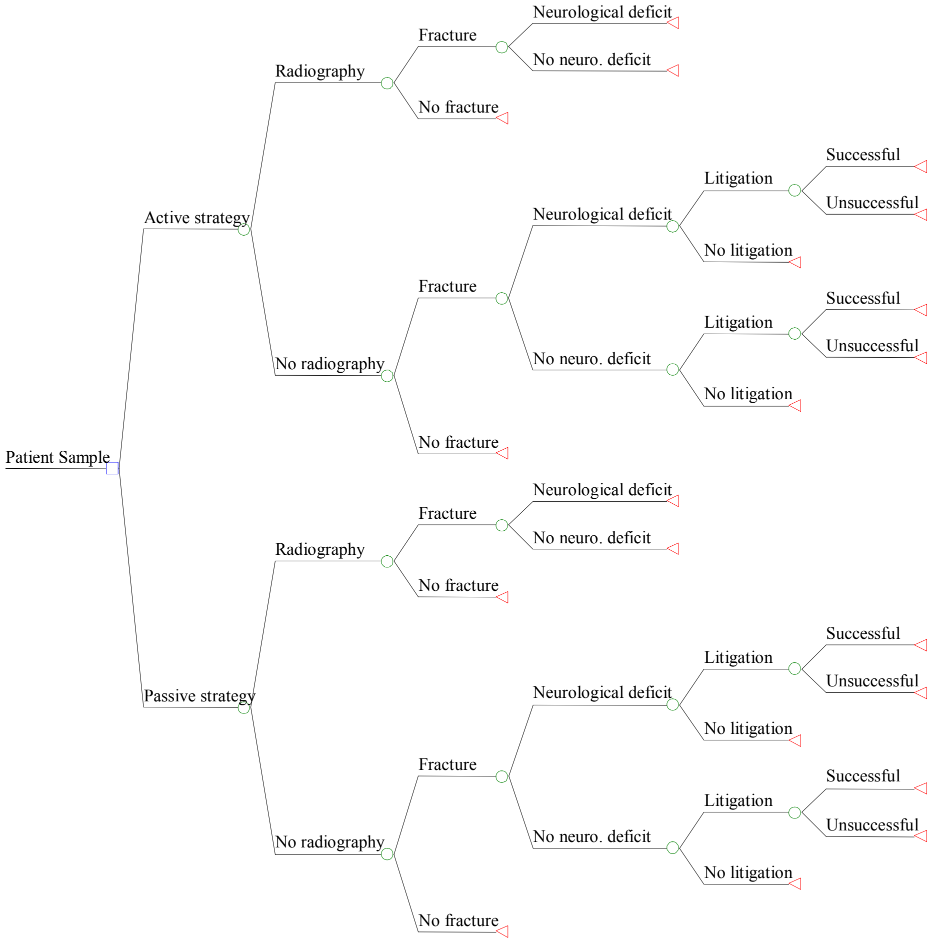

Study Design

For each terminal node within the decision tree, we will estimate the appropriate costs from both a health care and a societal perspective. Analysis will focus on identifying the cost savings attributable to the active dissemination strategy.

Results will be expressed in terms of expected values and 95% certainty intervals, obtained through probabilistic analysis. For each variable in the decision analysis a probability distribution will be derived either through use of confidence intervals and other data from clinical trials and the chart review, or though assumptions relating to the size of standard deviations associated with cost estimates.

For the probabilistic analysis, 10,000 replications will be conducted to yield 10,000 estimates of the outcome by re-running the model employing different values for each data point, randomly selected from that variables probability distribution.

Results will be expressed in terms of the net monetary benefit measure (NB). This is defined as:

NB $= \lambda \cdot (E_A - E_B) - (C_A - C_B)$

where

λ = threshold value for a unit of benefit
$(E_A - E_B)$ = incremental benefit of treatment A over treatment B
$(C_A - C_B)$ = incremental cost of treatment A over treatment B

Base analysis will assume a threshold value of a QALY of $50,000 with further analysis assuming a range from $10,000 to $100,000.

Source of Data

- Estimates of the sensitivity and specificity for the active strategy will be obtained from the proposed study.

- The cost of radiography will be obtained from both the Civic and General campuses of the Ottawa Hospital. We will obtain estimates of the costs of radiography form other participating centres which will allow estimation of a potential probability density function for costs.

- The amount of patient's time saved due to the reduction in testing will be derived from data obtained in the Phase 1 study. The value of this time will be valued by the mean wage rate.

- Incremental treatment for missed uncomplicated cervical spine fractures will be assumed to be an x-ray plus two additional physician consultations. The costs of the consultation will be obtained from the Ottawa Hospital and the OHIP Schedule of Fees and Benefits.

- Based on data from the Phase 1 and Phase 2 studies, it is assumed that no missed fractures will lead to neurological deficits.

- The rate and success of litigation for missed cervical spine fractures will be obtained from the Canadian Medical Protective Association.

- The cost of compensation packages for litigation settlement for missed cervical spine fractures will be obtained from the Canadian Medical Protective Association.

Analysis of Uncertainty

Analysis of uncertainty will focus assessing on the importance of individual parameter estimates in terms of the propagation of uncertainty from input values to the outcomes of interest. This form of analysis allows decision makers to identify those parameters which are of most important with respect to uncertainty concerning outcomes and thus allows prioritization for further research.

Three specific measures of importance will be adopted which will allow ranking of input parameters in terms of their contribution to uncertainty: expected value of perfect information, partial correlation coefficients and maximum separation distances.

Value of Information

Bayesian approaches to the analysis of uncertainty have focused on the estimation of the expected value of perfect information (EVPI) which is a measure of the reduction in opportunity loss associated with obtaining perfect information (no uncertainty) on a parameter (Felli and Hazen, Claxton). EVPI can be seen as both a measure of the sensitivity of results to the uncertainty around individual parameters as well as a means for identifying optimal use of scarce research dollars to reduce parameter uncertainty.

Two alternative formulations for EVPI have been suggested although a third formulation has been argued to be technically and theoretically superior. The formulations have been shown to give alternative absolute values for individual parameters but similar rankings. Analysis will provide estimates of individual parameter EVPI based on each formulation.

EVPI is based on the proportion of the probability density function where net benefit (NB) is negative. This can be expressed as:

$$EVPI = -\int_{-\varpi}^{0} f(NB)\,.\,NB\,dNB$$

Although in principle an exact method of calculation of EVPI is possible for certain cases, the design of a decision model typically does not allow such calculations in closed form. Instead, each formulation is based on the use of Monte Carlo simulation techniques to derive a set of estimates of net benefit. EVPI is estimated by taking the difference between the maximum NB over all treatment options for each replication and the NB from the optimum choice based on all replications:

$$EVPI = \Big(\sum_{r=1...R} \max_{i=1...n,Optimum} (EVPI_{ri}) - EVPI_{rOPTIMUM} \Big) / R$$

.

where

r	= replication number from the Monte Carlo simulation
k	= therapy
OPTIMUM	= therapy with the highest net benefit based on the average of all replictions

Each of the alternate formulations differ in assessing the impact of individual parameter uncertainty on the EVPI for the full model.

Felli and Hazen amongst others have suggested a formulation whereby EVPI is calculated in turn for each individual parameter (j) by assuming all other parameters fixed at their mean value. Claxton amongst others have suggested an alternative formulation where the EVPI for an individual parameter (j) is defined as the difference between EVPI when all variables are stochastic and the EVPI when the variable of interest is assumed constant at its mean value and all other variables remain stochastic. The third formulation builds on the approach of Claxton but instead of assuming the variable of interest is fixed at its mean value, EVPI is calculated by integrating alternate estimates of EVPI across the parameter's probability density function.

Partial Correlation Coefficients

Partial correlation coefficients assess the correlation between each input parameter and the outcomes of interest after controlling for other input values.

The partial correlation coefficient for an input (x_k) is assessed as follows.

1. Regression analysis is conducted whereby the dependent variable is the outcome (y) and the independent variables are the set of input values (X) except for input x_k. This allows estimation of coefficients $\hat{b}_o$ and $\hat{b}_j$. Estimates of the outcome are then calculated for each replication r based on these coefficients:

   $$\hat{y}_r = \hat{b}_0 + \sum_{j \neq k} \hat{b}_j . X_{j_r}$$

2. The above is repeated for variable x_k for each replication r.

   $$\hat{x}_{k\,r} = \hat{c}_0 + \sum_{j \neq k} \hat{c}_j . X_{j_r}$$

3. The difference between the estimated values for the variables ($\hat{y}_r$ and $\hat{x}_{k_r}$) and the replication values (y_r and x_{k_r}) are calculated.

   $y - \hat{y}$ and $x_k - \hat{x}_k$

4. The partial correlation coefficient for x_k is simply the Pearson correlation coefficient of $y-\hat{y}$ and $x_k-\hat{x}_k$

Maximum Separation Distances

The use of maximum separation distances has been termed Generalized Sensitivity Analysis and is technique for assessing the likelihood that a variable has influence over a binary decision. With respect to economic analysis, the binary decision relates to whether the expected net benefit of treatment is positive or negative.

To calculate the maximum separation distance for a variable x_k the following steps are required.

1. The model output from the Monte Carlo simulation is categorized by the forecasted net benefit (either positive or negative net benefits).

2. The vertical difference between cumulative probability distribution for the two categories is calculated for each value of x_k.

$$\left| \frac{R_{i0}}{R_0} - \frac{R_{i1}}{R_1} \right|$$

where

R_0 = number of replications with a negative net benefit
R_1 = number of replications with a positive net benefit
R_i = number of replications where $x_k \leq i$.

3. The maximum separation distance is simply the maximum value from step 2.

$$MSD(X_k) = \max_{i=-\varpi \ldots \varpi} \left| \frac{R_{i0}}{R_0} - \frac{R_{i1}}{R_1} \right|$$
