# Supplementary material for: A matched-pair cluster design study protocol to evaluate implementation of the Canadian C-spine rule in hospital emergency departments: Phase III
Source: Implement Sci. 2007 Feb 8;2:4. doi: 10.1186/1748-5908-2-4 (PMC1802999; doi:10.1186/1748-5908-2-4)
Supplement: Additional File 2 — Details of Sample size Calculation. The document provides a more detailed explanation of the variables and equation used to calculate the matched-pair cluster sample size. [file 1748-5908-2-4-S2.pdf]

Details of Sample Size Calculation

The study is based is on a complex design scheme using a stratified matched-pair cluster randomization design; that is, using: a stratification factor type of hospital (teaching vs community); a matched-pair (hospitals matched according to baseline cervical spine radiography); and cluster randomization (unit of randomization is the hospital and unit of analysis is the patient).

In general, as noted by Donner and Klar: [102]

Let: m_{1j} and m_{2j} denote the sizes of the clusters in the intervention and control groups respectively for the j-th matched-pair (j=1,2,…,k);

P_{1j} and P_{2j} denote the change in the before and after true cervical spine radiography event rates in the intervention and control groups respectively for the j-th matched-pair (j=1,2,…,k);

$\hat{P}_{1j}$ and $\hat{P}_{2j}$ denote the estimates of the change in the before and after true cervical spine radiography event rates P_{1j} and P_{2j} respectively and

$d_j = \hat{P}_{1j} - \hat{P}_{2j}$ the observed difference in the before to after change in the cervical spine radiography event rates for the j-th matched-pair (j=1,2,…,k).

Then the number of pairs k needed to achieve power $100(1-\beta)\%$ for detecting a difference Δ in the cervical spine radiography event rates at the two-sided $100(1-\alpha)\%$ significance level is:

$$k = (Z_{\alpha/2} + Z_{\beta})^2 \frac{Var(d_j)}{\Delta^2}$$

where
$$Var(d_j) = \frac{P_{1j}(1-P_{1j})}{m_{1j}} + \frac{P_{2j}(1-P_{2j})}{m_{2j}} + 2\sigma^2_{AM}$$

and σ^2_{AM} is the between-cluster variance component.

Note:
(1) For sample size calculation purposes, it is suggested: to replace P_{1j} and P_{2j} by their anticipated mean values P_1 and P_2; and set $m_{1j} = m_{2j} = m$.

(2) Additional pairs will need to be selected to adjust for the underestimation that results when the critical values from a normal distribution are used rather then the t-distribution. This correction as given by Snedecor and Cochran [123] is (f+3)/(f+1) where f is the number of degrees of freedom for the test.

From the Phase II Prospective Validation Study, the cervical spine radiography event rates for the participating ranged from 63.3% to 85.9% with an average of 76% and an annual accrual per hospital of approximately 400 patients; and an estimate of the between-cluster variance component is 0.00636 based on Gail et al [122] for the COMMIT trial (ie. $Var(d_j) = 0.0066125$). It is expected that the event rate in the control group will not change and for the intervention group a 15% relative decrease, or an absolute decrease of 11.4% from the baseline rate of 76%), in the event rate would be considered a minimal clinically important change based on a consensus of the ED clinical investigators (ie. $\Delta = 0.114$). Then for a significance level of 0.05 and power of 80%, $k = (1.96 + 0.84)^2 (0.0066125) / .114^2 = 3.9891$ and the number of matched-pairs required is $(7/5)(3.9891) = 5.5847$. Thus 6 matched-pair clusters will be required.

Lachin and Bautista [124] have indicated that the benefit from taking into account the stratification in calculating the sample size may be small unless there is considerable imbalance in prognostic factors. As noted by Donner and Klar [102], with the smaller effective sample size for the cluster randomization designs, this imbalance will tend to be larger and the benefits from stratification realized. However, since the exact benefits of stratifying by teaching and community hospitals in this matched-pair design are difficult to quantify, a conservative approach is adopted and the 6 matched-pair clusters will be selected.
